# *Anoxybacillus karvacharensis* sp. nov., a novel thermophilic bacterium isolated from the Karvachar geothermal spring in Nagorno-Karabakh

**DOI:** 10.1099/ijsem.0.005035

**Published:** 2021-10-01

**Authors:** Hovik Panosyan, Armine Margaryan, Nils-Kåre Birkeland

**Affiliations:** ^1^​ Department of Biochemistry, Microbiology and Biotechnology, Yerevan State University, Alex Manoogian 1, 0025, Yerevan, Armenia; ^2^​ Department of Biological Sciences, University of Bergen, NO-5020 Bergen, Norway

**Keywords:** *Anoxybacillus*, geothermal spring, next-generation sequencing, thermophiles, whole-genome sequencing

## Abstract

Twelve thermophilic *Anoxybacillus* strains were isolated from sediment and water samples from a Karvachar hot spring located in the northern part of Nagorno-Karabakh. Based on phenotypic, chemotaxonomic and phylogenetic characteristics, one of the isolates, designated strain K1^T^, was studied in detail. The cells are straight, motile rods that are 0.2–0.4×2.3–7.2 µm in size. The strain is a Gram-stain-positive, moderately thermophilic facultative anaerobe with an optimum growth temperature of 60–65 °C and a growth temperature range of 45–70 °C. Growth of strain K1^T^ was observed at pH 6–11 (optimum, pH 8–9) and was inhibited in the presence of NaCl concentrations above 2.5 % (optimum, 1–1.5 %). The isolate could utilize a wide variety of carbon sources, including d-arabinose, d-ribose, d-galactose, d-fructose, d-mannitol, maltose, aesculin, melibiose, sucrose, trehalose, raffinose, amidone, glycogen, turanose, d-lyxose, d-tagatose, potassium gluconate and 2-keto-gluconate. The strain was able to hydrolyse starch, casein and gelatin, was positive for oxidase and catalase, and reduced nitrate to nitrite, but was negative for H_2_S production. Production of urease and indole was not observed. The major cellular fatty acids were C_15 : 0_ iso, C_16 : 0_ and C_17 : 0_ iso (52.5, 13.6 and 19.6 % of total fatty acids, respectively). Strain K1^T^ shares >99 % 16S rRNA sequence similarity and a genomic average nucleotide identity value of 94.5 % with its closest relative, *Anoxybacillus flavithermus* DSM 2641^T^, suggesting that it represents a separate and novel species, for which the name *Anoxybacillus karvacharensis* sp. nov. is proposed. The type strain of *Anoxybacillus karvacharensis* is K1^T^ (=DSM 106524^T^=KCTC 15807^T^).

## Introduction

Thermophilic microbes have developed unique adaptations to high temperatures and represent important bioresources for thermostable enzymes and processes. Among the bacilli, representatives of the thermophilic genus *Anoxybacillus* are frequently isolated from terrestrial high-temperature habitats. The type species of the genus *Anoxybacillus *, *Anoxybacillus pushchinoensis* DSM 12423^T^, was first described by Pikuta *et al*. in 2000 [1]. Despite the name given to the genus, which suggests that they are microbes that thrive under anoxic conditions, many aerobic and aerotolerant or facultative anaerobic anoxybacilli have been isolated and described [2, 3]. The number of *Anoxybacillus* species has rapidly increased over the last decade and, at the time of writing, *Anoxybacillus* contains 23 validly described species and two subspecies [1, 4–22]. All species belonging to the genus *Anoxybacillus* are rod-shaped, spore-forming thermophiles, or moderate thermophiles, and share common ecological characteristics.

Two species, *Anoxybacillus contaminans* and *Anoxybacillus caldiproteolyticus* have been isolated from contaminated gelatin batches and sewage sludge, respectively [5, 15]. Another species, *Anoxybacillus tepidamans *, has been isolated from sugar beet extraction juice [15]. Several species have been isolated from heated soil samples. Two other species, *Anoxybacillus calidus* and *Anoxybacillus salavatliensis *, have been isolated from soil of thermal power plant and high-temperature well-pipeline sediment samples in Turkey [14, 20]. *Anoxybacillus geothermalis* has been isolated from mineral deposits in a geothermal station [18], while *Anoxybacillus amylolyticus* was isolated from geothermal soil located on Mount Rittmann in Antarctica [9].

The majority of *Anoxybacillus* species have been isolated from hot springs worldwide. *Anoxybacillus flavithermus* (* Anoxybacillus flavithermus* subsp. *flavithermus *) was isolated from a hot spring in New Zealand [1, 23], while *Anoxybacillus voinovskiensis* and *Anoxybacillus kamchatkensis* were isolated from a hot spring on the Kamchatka Peninsula in Russia [6, 8]. Biogeography and geological history have a strong influence on the structure of microbial diversity in geothermal springs. Many *Anoxybacillus* species have been isolated from geothermal springs located in different regions of the Alpine–Himalayan orogenic belt. *Anoxybacillus gonensis *, *Anoxybacillus ayderensis *, *Anoxybacillus kestanbolensis* and *Anoxybacillus kaynarcensis* were isolated from the Gonen, Ayder, Kestanbol and Kaynarca hot springs in Turkey, respectively [4, 7, 16]. *Anoxybacillus rupiensis* and *Anoxybacillus bogrovensis* were isolated from hot springs in the region of Rupi Basin and Dolni Bogrov in Bulgaria [10, 11]. *Anoxybacillus vitaminiphilus *, *Anoxybacillus eryuanensis* and *Anoxybacillus tengchongensis *, *Anoxybacillus sediminis *, as well as *Anoxybacillus flavithermus* subsp. *yunnanensis *, were isolated from hot springs in the regions of Puge, Eryuan, Tengchong, Tibet and Yunnan in China, respectively [13, 17, 19, 21]. *Anoxybacillus mongoliensis* [12] and *Anoxybacillus thermarum* [22] were isolated from the alkaline hot spring Tsenher, in Central Mongolia, and from thermal mud of the Euganean hot springs in Italy, respectively. Presumably, plate tectonics, geographic distance and isolation can cause biogeographical structuring, and drive speciation and evolution among prokaryotes, as previously shown for other terrestrial hot spring microbiota [24, 25]. Among the lesser-known high-altitude geothermal springs on Earth, the thermal springs located in the Lesser Caucasus mountain range are natural reservoirs of as-yet-undescribed microbial resources. These observations inspired us to explore the microbial diversity in the Armenian Highland, which is part of the Alpine–Himalayan orogenic belt [26]. In the present study, we report the isolation and complete polyphasic taxonomic characterization (including whole genome analysis and phylogenetic, chemotaxonomic, physiological and biochemical profiles) of a novel alkali-tolerant thermophilic, facultative anaerobic *Anoxybacillus* strain, K1^T^, which was isolated from a geothermal spring in Karvachar, Nagorno-Karabakh. Based on its genotypic and phenotypic properties, it is proposed that strain K1^T^ represents a novel species of the genus *Anoxybacillus *.

## Isolation and ecology

Combined water-sediment slurry samples were collected from a high-elevation geothermal spring at Karvachar (40° 17′ 41.00″ N, 46° 27′ 50.00″ E, at 1584 m altitude), Nagorno-Karabakh (Fig. S1, available in the online version of this article) in August 2016. Temperature, pH and conductivity were measured *in situ* using a portable combined pH/EC/TDS/ temperature tester (hanna HI98129/HI98130). The temperature of the sampling site was 70 °C. The spring water was circumneutral (pH 7.3) and showed relatively high mineralization (4600 μS cm^–1^). No specific permissions were required for the sample collection from the geothermal spring. All samples were kept in sterilized thermostatic flasks to maintain the habitat temperature and were transported to the laboratory. Before inoculation, all samples were pasteurized at 80 °C for 10 min to enrich for endospore-forming bacilli. Samples were enriched in nutrient broth (NB; Difco; pH 7.0) and incubated overnight with shaking at 250 r.p.m. and 60 °C. Turbid cultures were serially diluted and streaked onto nutrient agar (NA) plates to obtain separate colonies. All plates were incubated at 60 °C until visible colonies formed. Distinctive colonies were picked and subcultured by streaking onto the same medium several times to obtain pure isolates. The cultures were considered pure when only one morphological type of bacterium was observed by phase-contrast microscopy.

Twelve white/cream, smooth surface and circular colonies were selected for 16S rRNA sequence analysis, as described below, to determine their phylogenetic classifications. The 16S rRNA gene sequence similarities between the selected strains K1, K-1, K-33, K-35, K-80, K-83, K-97, K-98, K-99, K-QB2, KS-1 and KV-1 with those available in the GenBank database were between 98.0 and 99.9 % (Table S1). Because of its high amylase activity [26], strain K1 was selected for further characterization.

## 16S rRNA gene phylogeny

**Fig. 1.**
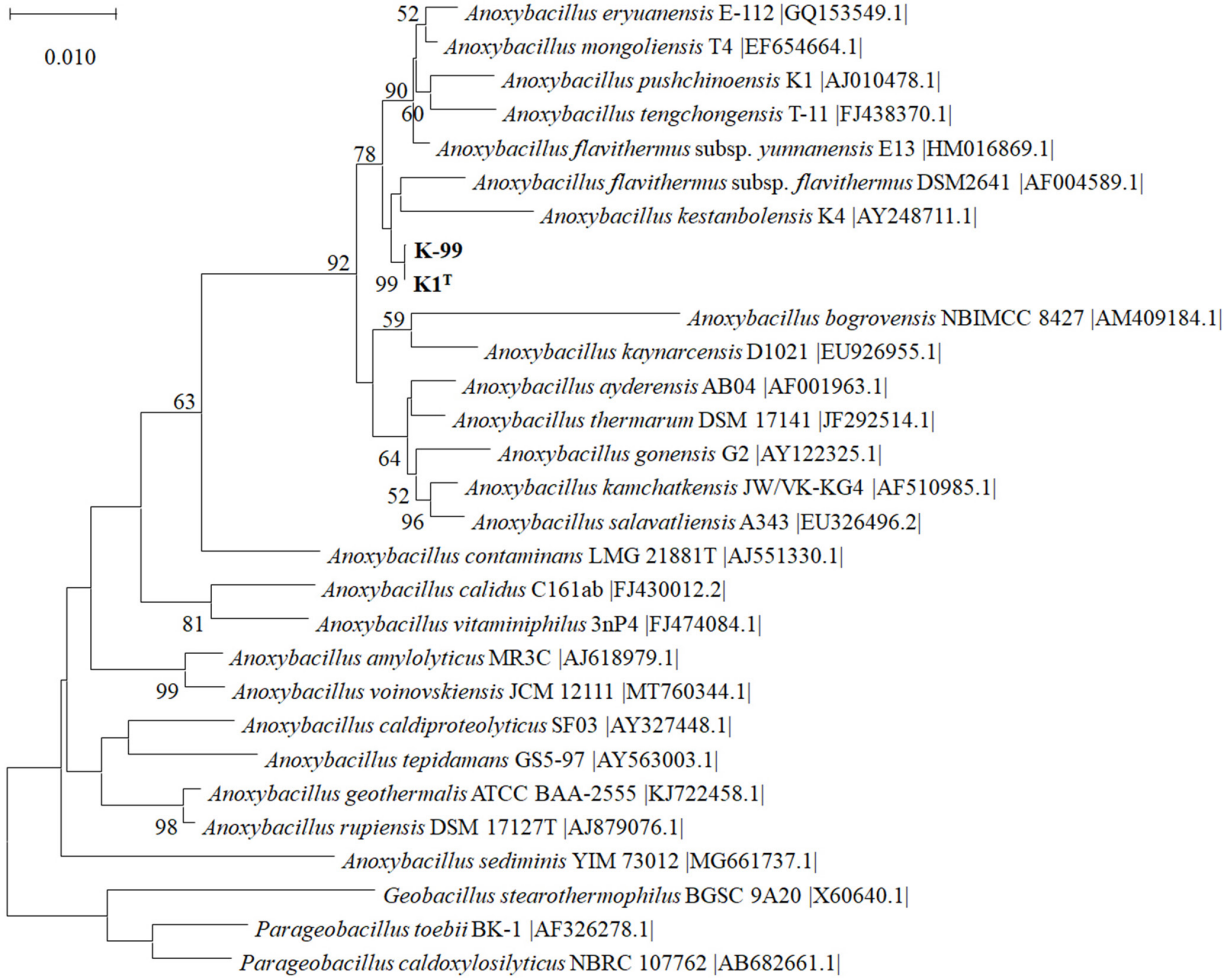
Phylogenetic tree based on 16S rRNA gene sequences of strains K1^T^ and K-99 (shown in bold) and representatives of all currently validly described species of the genus *Anoxybacillus *, inferred using the NJ method. Gaps and missing data were excluded. The optimal tree with the sum of branch length of 0.31395809 is shown. Accession numbers are shown in brackets. Bootstrap values (≥50 %) based on 1000 iterations are shown as percentages at the nodes. Bar, 0.01 nucleotide substitutions per site. Evolutionary analyses were conducted in mega X [28]. The tree was rooted with *Geobacillus* and *Parageobacillus* 16S rRNA gene sequences.

Genomic DNA was extracted from a pure culture using a GenElute Bacterial Genomic DNA Kit (Sigma-Aldrich), according to the manufacturer’s instructions. The 16S rRNA gene was selectively amplified using a universal bacterial primer set, 16SF (5′-GAGTTTGATCCTTGGCTCAG-3′) and 16 SR (5′-GAAAGGAGGTGATCCAGCC-3′) [27] corresponding to positions 27 to 1525 of the 16S rRNA gene of *Escherichia coli *. PCR reaction mixtures, with a final volume of 50 µl, contained 10 ng DNA, 10 µl 5×One*Taq* standard reaction buffer, 0.2 mM dNTP mix, 0.2 µM of each primer and 0.2 µl One*Taq* DNA polymerase (BioLabs). Amplification was performed using an initial denaturation at 94 °C for 5 min, followed by 30 cycles of denaturation at 94 °C for 30 s, annealing at 54 °C for 40 s, extension at 68 °C for 1 min, and a final extension at 68 °C for 5 min. The reaction was subsequently cooled to 4 °C. The PCR products were purified using the GenElute PCR Cleanup Kit (Sigma). Sanger sequencing of the amplicons was performed on an ABI PRISM capillary sequencer using the ABI Prism Big-Dye Terminator kit (Perkin Elmer) at the University of Bergen Sequencing Facility. The 16S rRNA gene sequence of strain K1^T^ was deposited in GenBank under the accession number MK418417, and was compared with available 16S rRNA gene sequences of cultured species deposited in GenBank using the blast algorithm. Phylogenetic analysis was performed using the neighbour-joining (NJ) and maximum-likelihood (ML) methods in mega X software [28]. Bootstrap analysis, based on 1000 replicates, was conducted to obtain confidence levels for the branches. The sequence of 1550 bases of the 16S rRNA gene of strain K1^T^ showed high sequence similarity to members of the genus *Anoxybacillus *. Strain K1^T^ had 99.81 % similarity to *A. flavithermus* DSM 2641^T^. A lower degree of similarity was found to other species of the genus *Anoxybacillus *. Strain K1^T^ is therefore a member of the genus *Anoxybacillus *, its closest relative being *A. flavithermus* DSM 2641^T^. The phylogenetic tree revealed distinct clustering of K1^T^ and K99, forming a separate lineage with high confidence with *A. flavithermus* DSM 2641^T^ and *A. flavithermus* subsp. *yunnanensis* DSM 23293 as closest neighbours (Fig. 1). In addition, phylogenetic analysis was performed using the ML method (Fig. S2), supporting the NJ-based branching order. Strain K99 was indistinguishable from K1^T^ at the 16S RNA gene sequence level, but differed from K1^T^ in that it was unable to utilize starch and casein. *A. flavithermus* DSM 2641^T^ was used as the reference strain for further analyses.

## Genome features

**Fig. 2.**
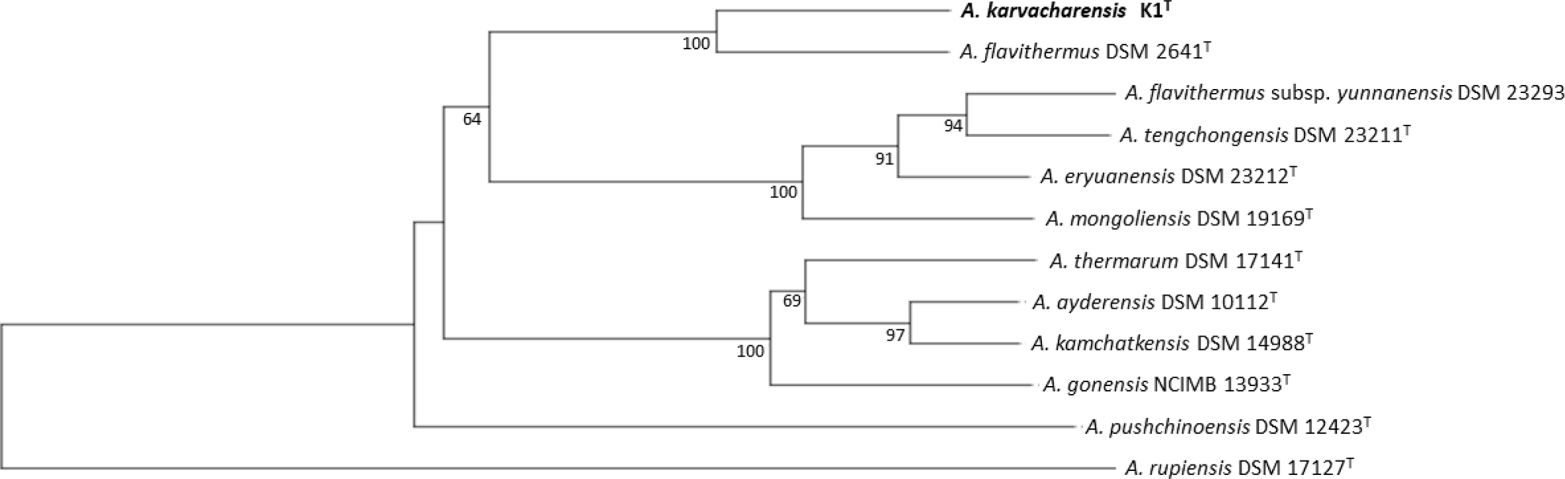
Phylogenomic tree of *A. karvacharensis* strain K1^T^ and related *Anoxybacillus* type strains. The tree was inferred with FastME 2.1.6.1 [34] from genome blast distance phylogeny (GBDP) distances calculated from genome sequences using the TYGS server (https://tygs.dsmz.de) [35]. The branch lengths are scaled in terms of GBDP distance formula d5. The numbers at branches are GBDP pseudo-bootstrap support values ≥64 % from 100 replications with an average branch support of 97.7 %. The tree was rooted at the midpoint [36]. Genome sequence accession numbers are as follows: *A. flavithermus *, CP020815.1; *A. flavithermus* subsp. *yunnanensis *, GCA_000753835; *A. tengchongensis *, JACHES000000000.1; *A. mongoliensis *, JACHEQ000000000.1; *A. pushchinoensis *, NZ_FOJQ00000000; *A. thermarum *, JXTH00000000.1; *A. ayderensis *, JXTG00000000.1; *A. kamchatkensis *, JACDUV000000000.1, *A. eryuanensis *, Gp0456347; *A. gonensis *, CP012152.1; *A. rupiensis *, Gp0401003.

**Fig. 3.**
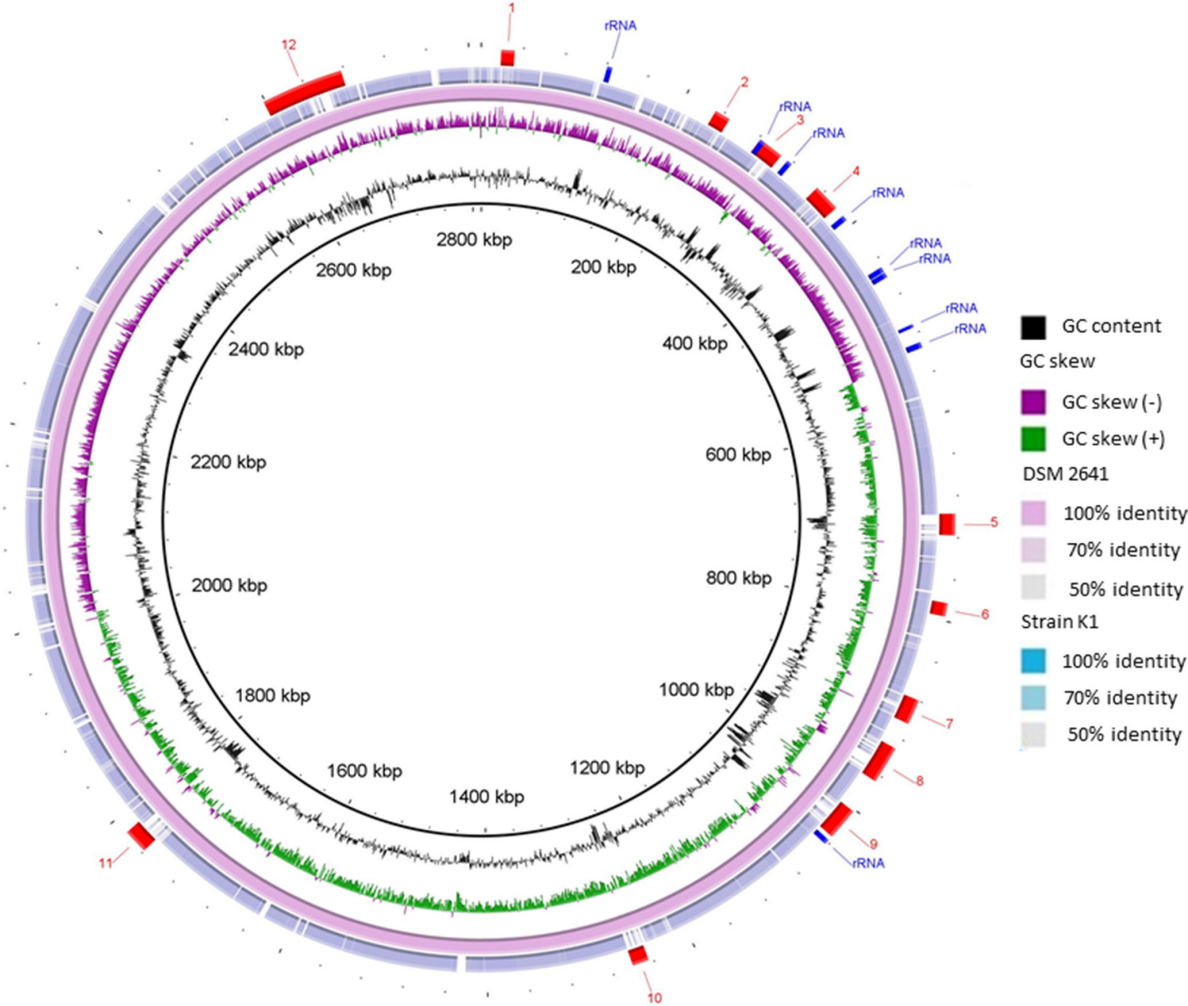
Circular representation of the genome of strain K1^T^ compared to *A. flavithermus* DSM2641^T^ as a reference. The rings were made using the blast Ring Image Generator (BRIG) server [37]. Rings from inside to outside: (1) G+C content (black); 2) GC skew (−, purple; +, green); (3) *A. flavithermus* strain DSM2641^T^; (4) *A. karvacharensis* strain K1^T^, (5) the position of the nine rRNA operons (blue arcs) and the 12 genomic islands (red arcs) in the reference strain genome. Genomic islands were predicted by using IslandViewer 4 (www.pathogenomics.sfu.ca/islandviewer/).

Whole-genome shotgun sequencing was performed using Illumina paired-end technology at Eurofins Genomics, Germany (www.gatc-biotech.com). The sequence data were assembled using CLC Genome Workbench software. Annotation was performed using rast (http://rast.nmpdr.org/) and the NCBI prokaryotic annotation pipeline (www.ncbi.nlm.nih.gov/genome/annotation_prok/). The whole-genome shotgun project has been deposited in GenBank under the accession number MQAD00000000. A draft genome sequence of approximately 2.7 Mb distributed onto 59 contigs was produced. The draft genome contained 2689 predicted-coding genes, 115 pseudogenes and two CRISPR arrays (Table 1), with a completeness of 99.34 % according to a CheckM analysis as implemented in KBase (https://www.kbase.us/). Strain K1^T^ shares 99.81 % 16S rRNA gene sequence similarity and a genomic average nucleotide identity (ANI) value of 94.5 % (two-way ANI), according to the ANI Calculator (http://enve-omics.ce.gatech.edu/ani/), with its closest relative, *A. flavithermus* DSM 2641^T^ (accession no. CP020815.1). Pairwise genome sequence similarities were calculated using the *in silico* Genome-to-Genome Distance Calculator and revealed 60.1 % overall genome similarity (using formula 2) between K1^T^ and strain DSM 2641^T^ (Table S2). These genomic DNA relatedness values are well below the threshold values recommended for the separation of novel bacterial species [29, 30]. For some species, e.g. *A. eryuanensis* vs. *A. tengchongensis* and *A. aydarensis* vs. *A. kamchatkensis* the pairwise digital DNA–DNA hybridization values were considerably higher (72.5 and 78.5 %, respectively). Strain K1^T^ can thus be considered as a separate species in the genus *Anoxybacillus *. This is also supported by a genome-based phylogenetic tree including all the closest genome neighbours (Fig. 2) showing a clustering of strain K1^T^ and DSM 2641^T^ and confident separation from all other *Anoxybacillus* species. A circular representation of the genome of strain K1^T^ compared with that of *Anoxybacillus flavithermus* DSM2641^T^ is shown in Fig. 3 and shows a large number of scattered small non-homologous regions, some of which represent genomic islands and strongly G+C biased regions in the reference strain, commonly indicating laterally transferred genes. The G+C content of strain K1^T^ was 41.6 mol%, which is within the 41.4–42.0 mol% range of its closest relatives in Fig. 2.

**Table 1.** Genome features and statistics of strain K1^T^

GenBank accession	MQAD00000000
No. contigs	59
No. base pairs	2 722 200
Genome coverage	1500×
G+C content (mol%)	41.6
No. rRNAs	5, 4, 1 (5S, 16S, 23S)
No. tRNAs	64
No. genes (total)	2883
No. CRISPRs	Two arrays, 68 spacers

*Assessed by CRISPRCasFinder (https://crisprcas.i2bc.paris-saclay.fr/CrisprCasFinder/Index).

## Morphology, physiology and chemotaxonomy

After 24 h of growth on NA, colonies of strain K1^T^ were 2–3 mm in diameter, cream-coloured, smooth and circular with regular round edges. K1^T^ did not form pigmented (yellow) colonies, unlike *A. flavithermus* DSM 2641^T^, when cultivated on agar or in liquid NB medium (Fig. S3), suggesting a lack of carotenoids. The morphological properties of strain K1^T^ were determined using light microscopy and scanning electron microscopy. In the exponential growth phase, most of the cells occurred singly. Endospore formation was verified by light microscopy in both liquid and solid media. Gram-positive staining was confirmed using standard Gram’s reaction. Motility was observed using oil immersion phase-contrast microscopy. The rod-shaped cells were 2.3–7.2 µm long and 0.2–0.4 µm wide with ellipsoidal subterminal endospores located in swollen sporangia (Fig. S4). The motility of strain K1^T^ was observed by phase-contrast microscopy and was confirmed by the identification of flagellar gene clusters (in total, 39 operons and regulatory genes distributed within contigs 8, 11, 24 and 31).

The physiological and biochemical characteristics of the isolate were investigated as described by Smibert and Krieg [31]. Each experiment was performed in triplicates. The range and optimal values of growth at different temperatures, pH values and salinities were determined by measuring the optical density spectrophotometrically at *λ*=540 nm. The temperature range for growth was determined by incubating the strain in NB at 5 °C temperature intervals, ranging from 30 to 80 °C with shaking. The effect of pH on growth was determined at optimal growth temperature in the pH range 5.0–12.0, with 0.5 pH increments. The pH values of the media were adjusted with either 1 M NaOH or 1 M HCl at room temperature. Strain K1^T^ grew between 45–70 °C, with optimal growth at 60–65 °C, and at pH 6.0–11.0, with optimum growth at pH 8–9. The growth characteristics indicated that strain K1^T^ is an alkali-tolerant moderate thermophile. The effect of salinity was also determined by growing K1^T^ in the medium containing 0.5 % peptone, 0.5 % yeast extract and supplemented with 0.5–5 % (w/v) NaCl at intervals of 0.5%, compared to a control broth without NaCl supplementation, and at optimal growth temperature and pH. The growth of strain K1^T^ was optimal at 1–1.5 % NaCl and was inhibited at NaCl concentrations above 2.5 %. Growth occurred in the absence of NaCl.

Anaerobic growth was observed in NA stab culture tubes incubated at optimal growth conditions (60 °C, pH 8.0) and covered with a paraffin film to reduce oxygen diffusion. Growth under strict anaerobic conditions in a serum flask with a mineral salt freshwater medium supplemented with 0.2 % glucose (w/v) and reduced with sulfide was also demonstrated. Based on its ability to grow under both anaerobic and aerobic conditions, strain K1^T^ can be classified as a facultative anaerobe. The shortest doubling time for the strain was 30 min under optimal conditions for growth.

The ability to utilize various substrates (glucose, arabinose, mannitol, maltose, sucrose, lactose, ribose, galactose, fructose, xylose, sorbitol and citrate) as carbon and energy sources was examined in a minimal liquid salt medium consisting of (g l^–1^): K_2_HPO_4_, 2.6; KH_2_PO_4_, 0.8; KNO_3_, 5.0; NaCl 5.0; and MgSO_4_, 0.5. Carbohydrates were added at a final concentration of 1 % (w/v). Gas production and acid formation were tested using a medium containing (g l^−1^): carbohydrates, 10; (NH_4_)_2_HPO_4_, 1; KCl, 0.2; MgSO_4_·7H_2_O, 0.2; and yeast extract, 0.2, with the addition of 15 ml of a 0.04 % (w/v) solution of bromocresol purple. In addition, the use of carbon sources was assessed using an API 50 CHB strip (bioMerieux).

The strain was positive for gas and acid formation from d-glucose and could utilize a wide range of carbon sources including d-arabinose, d-ribose, d-galactose, d-fructose, d-mannitol, maltose, aesculin, melibiose, sucrose, trehalose, raffinose, amidone, glycogen, turanose, d-lyxose, d-tagatose, potassium gluconate and 2-keto-gluconate (Tables 2 and S3). The following carbon sources were not assimilated according to the API 50 CHB strips: glycerol, erythritol, l-arabinose, d-xylose, l-xylose, d-adonitol, d-mannose, l-sorbose, l-rhamnose, dulcitol, inositol, d-sorbitol, methyl α-d-mannopyranoside, methyl α-d-glucopyranoside, *N*-acetylglucosamine, amygdalin, arbutin, salicin, cellobiose, lactose, inulin, d-melezitose, xylitol, gentiobiose, d-fucose, l-fucose, d-arabitol, l-arabitol and 5-keto-gluconate (Table S3).

**Table 2.** Differential phenotypic characteristics of strain K1^T^ and related species from the genus *Anoxybacillus
* Strain: 1, K1^T^; 2, *A. flavithermus* subsp. *flavithermus* DSM 2641^T^ [1]; 3, *A. flavithermus* subsp. *yunnanensis* KCTC 13759^T^ [19]; 4, *A. gonensis* NCIMB 13971^T^ [4]; 5, *Anoxybacillus, salavatliensis* DSM 22626^T^ [20]; 6, *A. kamchatkensis* JW/VK-KG4^T^ [8]; 7*, A. tengchongensis* KCTC 13721^T^ [13]; 8, *A. eryuanensis* KCTC 13720^T^ [13]; 9, *A. pushchinoensis* DSM 12423^T^ [1, 38]. *A. flavithermus* DSM 2641^T^ was used as a reference strain. FA, facultative anaerobe; A, aerobe; AA, aerotolerant anaerobe; E, ellipsoidal; S, spherical; O, oval; T, terminal; ST, subterminal; VP, Voges–Proskauer; +, positive; −, negative; nd, not detected or no data.

Characteristics	1*	2*	3	4	5	6	7	8	9
Size (µm), long/wide	2.3–7.2/ 0.2–0.4	2.3–7.1/ 0.85	1.2–7.0/ 0.4–0.7	5.0/ 0.75	2.34/ 0.71	2.5–8.8/1.0	4.5–5.5/ 0.6–1.2	4.5–4.7/ 0.5–0.7	2.5–3.0/ 0.4–0.5
Motility	+	+	+	+	+	+	+	+	−
Spore shape	E	S	E	S	E	O	E	E	S
Spore location	ST	T	T	T	T	T	T	T	T
O_2_ requirement	FA	FA	FA	FA	FA	FA	FA	FA	FA/AA
Temperature range (°C) (optimum)	45–70 (60–65)	30–72 (60)	30–66 (60)	40–70 (55–60)	37–69 (60)	37–66 (57–62)	30–75 (50)	35–70 (55)	37–65 (62)
pH range (optimum)	6.0–11.0 (8.0–9.0)	6–9.0 (7.0)	5.5–10.0 (7.0–7.5)	6.0–10.0 (7.5–8.0)	6.0–10.0 (8.0)	5.7–9.9 (6.8–8.5)	7.0–11.0 (8.5)	7.0–11.0 (8.0)	8.0–10.5 (9.5–9.7)
NaCl tolerance (%, w/v) (optimum)	2.5 (1.0–1.5)	2.5 (0.5)	3.5 (0.3)	4 (2.0)	4.5 (2)	nd (nd)	4 (1.5)	3 (0.5)	3 (0.5–1.0)
Catalase	+	+	+	+	+	−	+	+	−
Oxidase	+	+	nd	+	+	−	+	+	nd
VP test	−	+	nd	nd	−	nd	nd	nd	nd
Nitrate reduction	+	+	−	+	+	nd	+	−	+
Utilization of:									
d-Glucose	+	+	+	+	+	+	+	+	+
l-Arabinose	−	+	+	−	−	−	nd	nd	−
d-Mannitol	+	+	+	+	+	+	+	+	−
Maltose	+	+	+	nd	+	+	+	+	nd
d-Ribose	+	+	−	nd	nd	−	nd	nd	−
d-Galactose	+	−	+	nd	+	+	nd	nd	−
d-Fructose	+	−	−	nd	+	+	+	+	+
d-Xylose	−	−	+	+	−	−	−	−	−
d-Sorbitol	−	+	nd	nd	−	−	nd	nd	−
Hydrolysis of:									
Starch	+	+	−	+	+	−	+	+	+
Casein	+	+	nd	−	−	−	nd	nd	−
Gelatin	+	−	−	+	+	−	+	+	−

*Data obtained in this study.

A range of hydrolytic enzyme activities was tested. Starch hydrolysis was tested by flooding cultures onto solid enrichment medium containing 0.2 % (w/v) starch with Lugol’s iodine solution (2 % KI and 1 % I_2_ in dH_2_O). For assessing casein hydrolysis, a solid minimal medium with an equal quantity of skimmed milk was used. For assessing gelatin hydrolysis, 1 % (w/v) gelatin was used in minimal medium. Lipolytic activity was assessed using a minimal medium containing 1 % Tween 40 (v/v) as the carbon source. Strain K1^T^ was positive for gelatin liquefaction and hydrolysis of casein and starch but was negative for lipolytic activity. Catalase activity was tested using 3 % (w/v) H_2_O_2_ by assessing bubble production. Oxidase activity was assessed using 1 % (w/v) *N*,*N*,*N*,*N*-tetramethyl-*p*-phenylenediamine. The strain was both catalase- and oxidase-positive. Nitrate reduction to nitrite, Voges–Proskauer (VP) reaction, production of H_2_S and indole, utilization of citrate, β-galactosidase, arginine dihydrolase, lysine decarboxylase, ornithine decarboxylase, urease and tryptophan deaminase activities were tested using an API 20 E strip, according to the manufacturer’s instructions (bioMérieux). The strain was capable of reducing nitrate to nitrite but was negative for the VP reaction. The strain was not able to produce urease, H_2_S, or indole. According to the API 50 CHB strip, the strain was negative for arginine dihydrolase, lysine decarboxylase, ornithine decarboxylase and tryptophan deaminase, but positive for β-galactosidase. The reference strain *A. flavithermus* DSM 2641^T^ was positive for arginine dihydrolase, β-galactosidase, lysine decarboxylase and tryptophan deaminase, but negative for ornithine decarboxylase [23].

Although strain K1^T^ shared common properties with other members of the genus *Anoxybacillus *, it was distinguishable from its closest relatives by certain phenotypic characteristics. The specific physiological properties differentiating strain K1^T^ from its most closely related type strain, *A. flavithermus* (* A. flavithermus* subsp. *flavithermus *) DSM 2641^T^, include: (1) lack of pigmentation, (2) endospore shape and location (ellipsoidal subterminal), (3) a more restricted temperature range for growth, (4) alkaline-tolerance, (5) β-galactosidase positivity, (6) spectrum of carbon and nitrogen source utilization (e.g. hydrolysis of gelatin), and (7) a negative VP test (Table 2).

According to the API 50 CHB strip, the two strains differed in the use of 17 carbon sources (Table S3). Strain K1^T^ also differs from *A. flavithermus* subsp. *yunnanensis* by cell size, endospore shape and location, more restricted temperature and pH range for growth, NaCl tolerance, ability to reduce nitrate, and to hydrolyse starch and casein (Table 2). A disc diffusion test was performed on NA plates to determine the antimicrobial susceptibility of strain K1^T^. Antimicrobial susceptibility test discs (HiMedia) of the following antibiotics were used: ciprofloxacin (5 µg), doxycycline (30 µg), erythromycin (15 µg), ampicillin (10 µg), kanamycin (30 µg), gentamycin (10 µg), tetracycline (30 µg), streptomycin (10 µg), penicillin (6 µg), trimethoprim (5 µg), imipenem (10 µg), furadonin (300 µg), ceftriaxone (30 µg), cefoxitin (30 µg), amoxicillin/clavulanic acid (20/10 µg) and sulfamethoxazole (100 µg). The strain was sensitive to all antibiotics tested.

Cellular fatty acid composition was determined at the Leibniz Institute DSMZ-German Collection of Microorganisms and Cell Cultures. Fatty acid methyl esters (FAMEs) were obtained from freeze-dried cell pellets by saponification, methylation and extraction using a previously described method with minor modifications [32, 33]. The FAME mixtures were analysed using the Sherlock Microbial Identification System (midi, Microbial ID), which consists of an Agilent model 6890 N gas chromatograph fitted with a 5 % phenyl-methyl silicone capillary column (0.2 mm××25 m), a flame ionization detector and an Agilent model 7683A automatic sampler, and then identified using the midi database. Peaks were automatically integrated, and fatty acid names and percentages were calculated using MIS Standard Software (Microbial ID). The gas chromatographic parameters were as follows: carrier gas, ultra-high-purity hydrogen; column head pressure, 60 kPa; injection volume, 2 µl; column split ratio, 100 : 1; septum purge, 5 ml min^−1^; column temperature, 170–270 °C at 5 °C min^−1^; injection port temperature, 240 °C; and detector temperature, 300 °C. The fatty acid analysis revealed that strain K1^T^ synthesized mainly iso- and anteiso-branched saturated fatty acids, similar to other thermophilic *Bacillus* and *Geobacillus* species. The FAME profiles of K1^T^ showed that the main fatty acids were C_15 : 0_ iso (52.02 %), followed by C_17 : 0_ iso (19.09 %) and C_16 : 0_ (13.15 %) (Table S4). Iso-branched saturated fatty acids were dominant, accounting for more than 84 % of the total cellular fatty acids. This finding is in agreement with the general features of the fatty acid profiles of recognized *Anoxybacillus* species. The contents of fatty acids with longer chains for strain K1^T^ were as follows: C_16 : 0_ (13.15 %), C_16 : 0_ iso (3.83 %), C_17 : 0_ (0.48 %), C_17 : 0_ iso (19.09 %) and C_17 : 0_ anteiso (4.62 %). However, lower amounts of shorter fatty acids, such as C_14 : 0_ (1.90 %), C_14 : 0_ iso (0.25 %), C_15 : 0_ (0.4 %), C_14 : 0_ iso 3-OH (0.31 %) and C_15 : 0_ anteiso (2.18 %) were also observed. In contrast to other *Anoxybacillus* species, strain K1^T^ contained low amounts of C_14 : 0_ iso 3-OH (0.31 %) and C_18 : 1_ *ω*9*c* (1.01 %).

According to the 16S rRNA gene sequence analysis, strain K1^T^ is closely related to *A. flavithermus* DSM 2641^T^ and *A. kestanbolensis* NCIMB 13971^T^. DNA–DNA relatedness and phenotypic differences indicate that the isolate is distinct from these related strains, and therefore represents a novel species of the genus *Anoxybacillus *. Therefore, we propose the name *Anoxybacillus karvacharensis* sp. nov. for this novel isolate.

## Protologue

Based on the results of morphological, physiological chemotaxonomic and phylogenetic analyses, strain K1^T^ belongs to the genus *Anoxybacillus* and represents a novel species, for which the name *Anoxybacillus karvacharensis* sp. nov. is proposed.

## Description of *Anoxybacillus karvacharensis* sp. nov.

*Anoxybacillus karvacharensis* [kar.va.cha.ren´ sis. N.L. masc. adj. *karvacharensis*, pertaining to Karvachar, which is a geothermal spring in the province of Shahumyan, Nagorno Karabakh (Republic of Artsakh), where the type strain was isolated].

Cells are Gram-stain-positive, rod-shaped, occurring singly or in short chains, motile and 0.2–0.4×2.3–7.2 µm in size. Subterminal ellipsoidal endospores were formed. Colonies are 2–3 mm in diameter, cream-coloured, and regular in shape with round edges. The strain is moderately thermophilic, facultatively anaerobic and alkali-tolerant. The temperature range for growth is 45–70 °C, with an optimum temperature range of 60–65 °C. The pH range for growth is pH 6.0–11.0, with an optimal pH of 8.0–9.0. Growth occurs at NaCl concentrations of up to 2.5 %. The strain is catalase- and oxidase-positive. The isolate is able to utilize a wide variety of carbon sources including d-arabinose, d-ribose, d-galactose, d-fructose, d-mannitol, maltose, aesculin, melibiose, sucrose, trehalose, raffinose, amidone, glycogen, turanose, d-lyxose, d-tagatose, potassium gluconate and 2-keto-gluconate. Starch, casein and gelatin are hydrolysed by the strain, but Tween 40 is not. Nitrate is reduced to nitrite, but the strain is negative for H_2_S production. The strain is negative in the VP test. Urease, dihydroxyacetone and indole are not produced. Arginine di-hydrolase, lysine decarboxylase, ornithine decarboxylase and tryptophan deaminase tests are negative, but the β-galactosidase test is positive. The fatty acid profile contains C_15 : 0_ iso, C_16 : 0_ and C_17 : 0_ iso as major components. Growth is inhibited in the presence of ciprofloxacin (5 µg), doxycycline (30 µg), erythromycin (15 µg), ampicillin (10 µg), kanamycin (30 µg), gentamycin (10 µg), tetracycline (30 µg), streptomycin (10 µg), penicillin (6 µg), trimethoprim (5 µg), imipenem (10 µg), furadonin (300 µg), ceftriaxone (30 µg), cefoxitin (30 µg), amoxicillin/clavulanic acid (20/10 µg) and sulfamethoxazole (100 µg).

The genomic G+C content of K1^T^ is 41.6 mol%. The type strain, K1^T^ (=DSM 106524^T^=KCTC 15807^T^), was isolated from the Karvachar geothermal spring, Nagorno Karabakh.

## Supplementary Data

Supplementary material 1
