# Supplementary material for: Anoxybacillus karvacharensis sp. nov., a novel thermophilic bacterium isolated from the Karvachar geothermal spring in Nagorno-Karabakh
Source: Int J Syst Evol Microbiol. 2021 Oct 1;71(10):005035. doi: 10.1099/ijsem.0.005035 (PMC8604165; doi:10.1099/ijsem.0.005035)
Supplement: Supplementary material 1 [file ijsem-71-5035-s001.pdf]

Anoxybacillus karvacharensis sp. nov., a novel thermophilic bacterium isolated from the Karvachar geothermal spring in Nagorno Karabakh

Hovik Panosyan, Armine Margaryan, Nils-Kåre Birkeland

SUPPLEMENTARY FIGURES AND TABLES

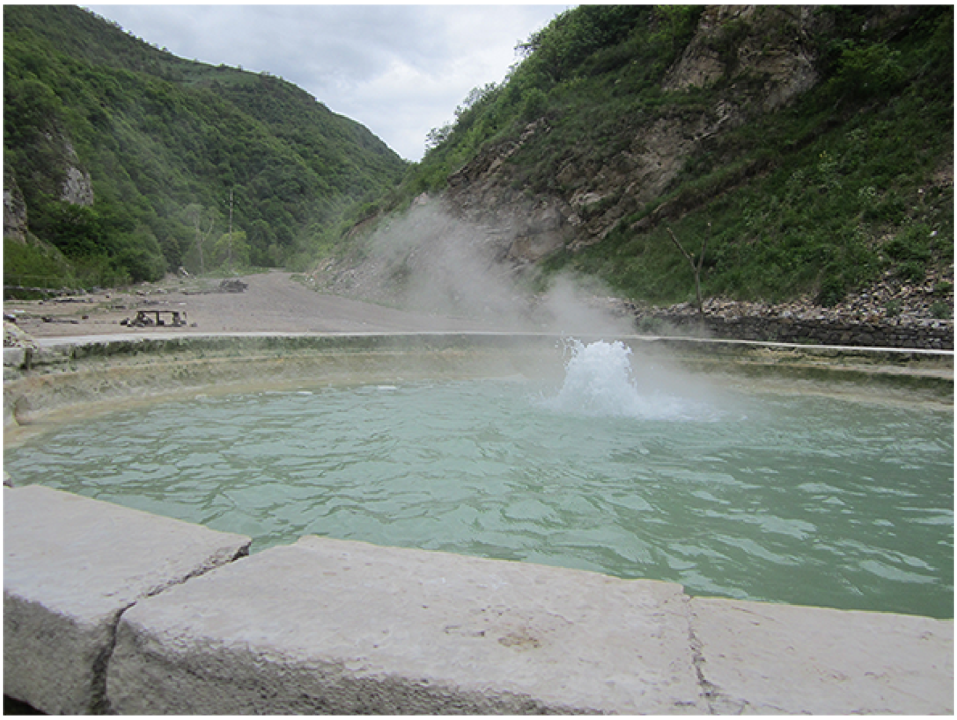

Fig. S1. High-elevation geothermal spring at Karvachar, Nagorno Karabakh, home habitat of strain K1^{T}.

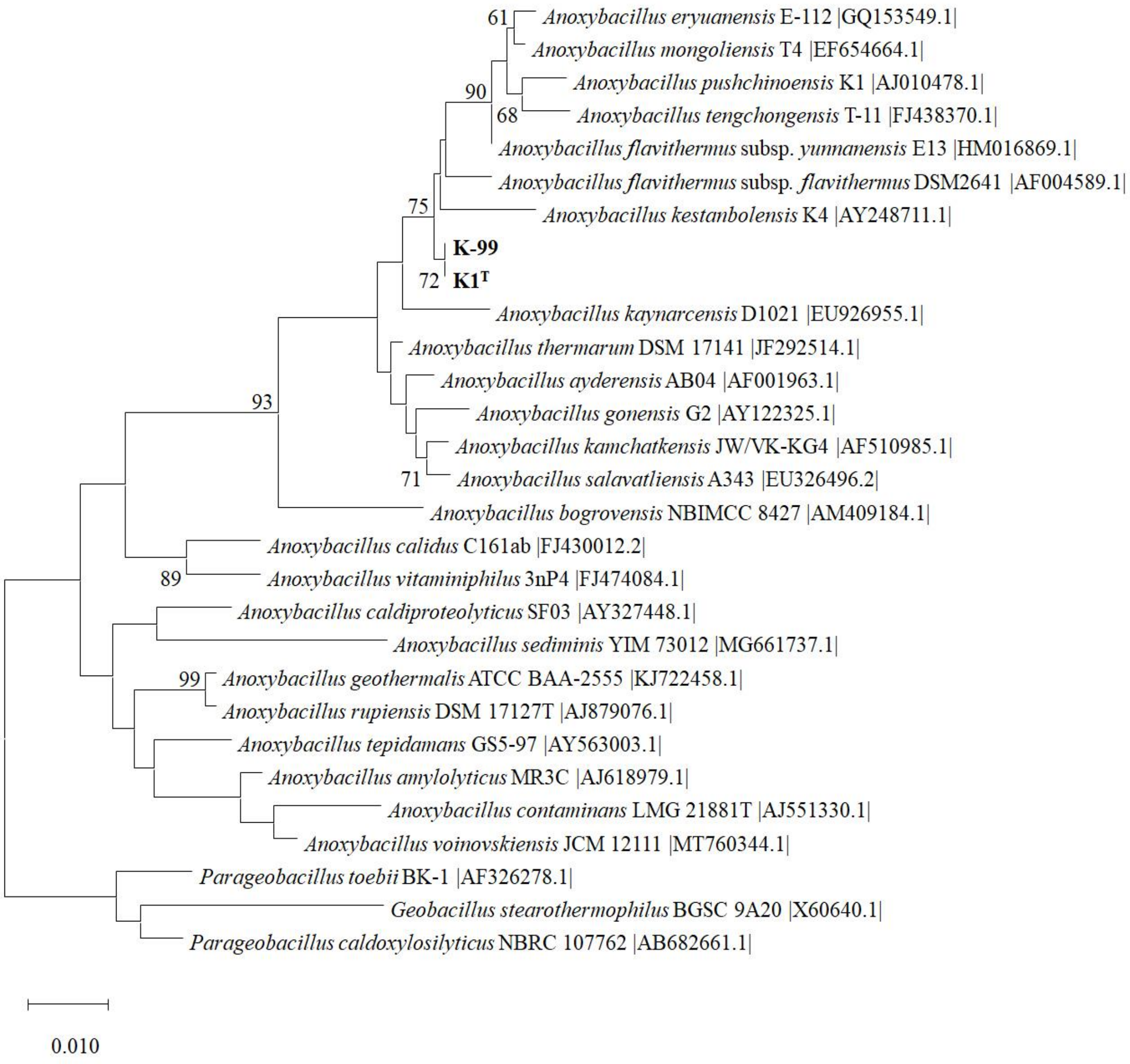

Fig. S2. Phylogenetic tree based on 16S rRNA sequences of strain $K1^T$ and representatives of all currently valid species of the *Anoxybacillus* genus, as well as species of close related genera *Gebacillus* and *Parageobacillus,* inferred using the Maximum Likelihood method and Tamura-Nei model. Gaps and missing data were excluded. The tree with the highest log likelihood (-5466.77) is shown. Accession numbers are shown in brackets. Bootstrap values (≥50%) based on 1000 iterations are shown as percentages at the nodes. Bar, 0.01 nucleotide substitutions per site. Evolutionary analyses were conducted in MEGA X.

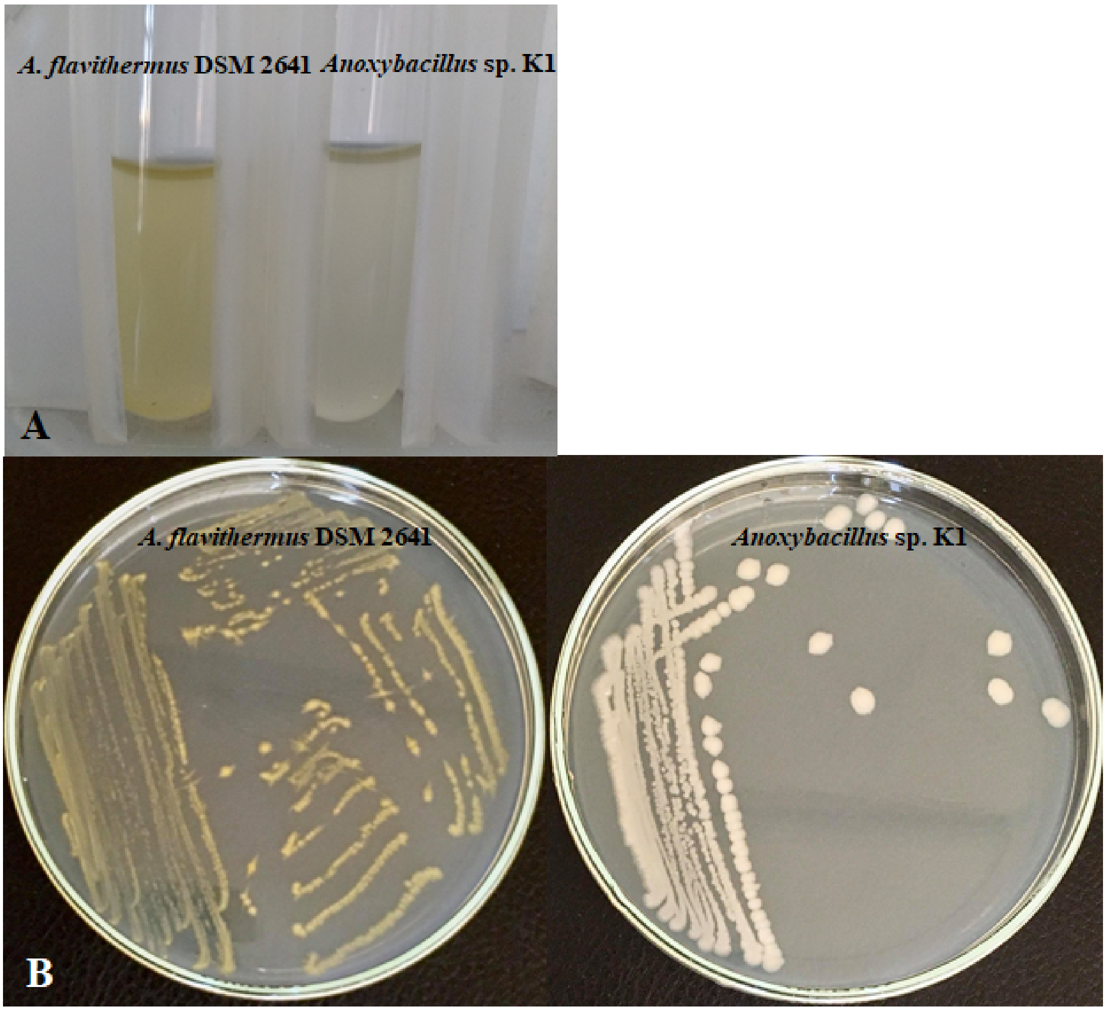

Fig. S3. Colonies and pigmentation of strain K1^T and the reference *Anoxybacillus flavithermus* DSM2641^T type strain. A, in tubes after 48 hours cultivation in NB. B, Colonies on NB agar plates.

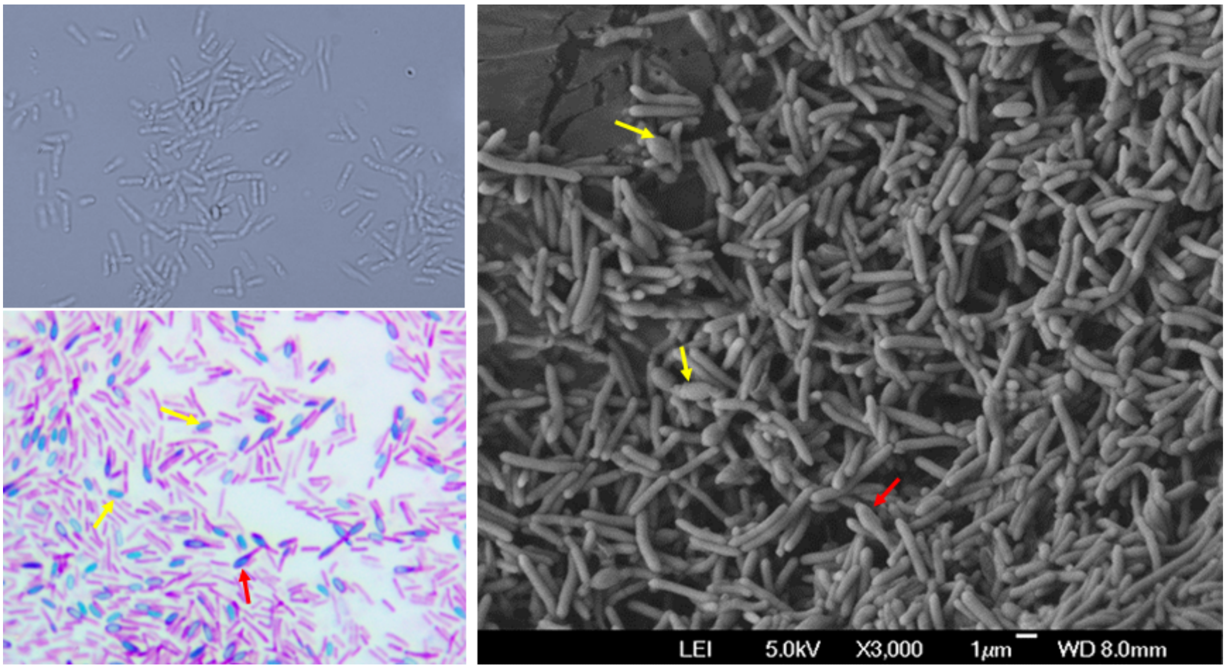

Fig. S4. Light micrographs (×1000, OMAX) and scanning electron micrograph of cells of strain K1^T (24 h cultivation) showing typical rod shape cells occurring singly. Subterminal endospore formation can be seen as swollen ends (red arrow). Free ellipsoidal spores are also depicted (yellow arrows). Spore staining was performed by Schaeffer-Fulton`s method using malachite green and safranin stains [1]. As the result spores stains in blue-green, bacteria in red color. Scanning electron microscopy (SEM) was performed using a Jeol JSM-7400F scanning electron microscope at the Molecular Imaging Center Platform at the University of Bergen (https://www.uib.no/en/rg/mic). A sample of the bacterial culture was fixed with 2.5 % glutaraldehyde for at least 1 h. After 3 washes in 0.1 M phosphate buffer, pH 7.2, post-fixation in 1 % osmium tetroxide was done for 1 h. Dehydration was performed successively in 30%, 50 %, 90 % and 2 x 100 % ethanol, followed by filtration of the cells onto a 0.2 µm black polycarbonate filter. Pieces of the filter were critical-point dried for at least 1 h, mounted onto a SEM stub and sputter-coated with 10 nm gold/palladium particles.

Table S1. BLAST results of 16S rRNA gene sequences of thermophilic anoxybacilli isolates and their accession numbers

Isolate	Sequence length (bp)	Closest match, phylotype accession no.	% Similarity to closest match	Accession no.
K-80	1463	*A. flavithermus* KC503890	99.3	MK418410
K-83	1462		99.6	MK418411
K-97	1475		99.8	MK418412
K-98	1466		99.4	MK418413
KS-1	1453		99.5	MK418418
KV-1	839		98.0	MK418419
K-1	1472		99.9	MK418420
K-33	1461		99.4	MK418427
K-35	1468		99.2	MK418428
K-QB2	1443		99.1	KY203976
K-99	1447	*A. flavithermus* FJ950739	99.9	MK418414
**K1^T**	1550	*A. flavithermus* P021838	99.9	MK418417

Table S2. Genome-to-genome distance analysis of strain K1 and related *Anoxybacillus* spp. using the Genome-to-Genome Distance Calculator [2] as implemented at the TYGS server (https://tygs.dsmz.de). The figures indicate pairwise dDDH (d_4) values in percentages.

	1	2	3	4	5	6	7	8	9	10	11	12
1. *A. karvacharensis* K1	100											
2. *A. flavithermus* DSM 2641^T	60.1	100										
3. *A. mongoliensis* DSM 19169^T	37.4	38.1	100									
4. *A. eryuanensis* DSM 23212^T	36.5	36.5	63.0	100								
5. *A. flavithermus* subsp. *yunnanensis* DSM 23293	36.3	36.4	53.4	67.5	100							
6. *A. tengchongensis* DSM 23211^T	35.8	36.3	53.5	68.5	72.5	100						
7. *A. pushchinoensis* DSM 12423^T	35.7	32.2	30.5	30.4	31.4	30.6	100					
8. *A. gonensis* NCIMB 13933^T	34.7	35.1	34.6	33.4	33.4	33.3	30.9	100				
9. *A. ayderensis* DSM 10112^T	34.5	35.1	34.6	33.6	33.8	33.4	31.9	58.2	100			
10. *A. thermarum* DSM 17141^T	34.2	34.6	33.7	33.1	32.9	32.8	32.0	55.2	60.5	100		
11. *A. kamchatkensis* DSM 14988^T	33.6	34.1	34.4	33.5	33.4	33.5	30.5	59.1	78.5	62.2	100	
12. *A. rupiensis* DSM 17127^T	20.8	22.9	19.7	19.4	20.1	19.8	20.0	21.6	21.1	21.3	18.7	100

Table S3. Utilization of different carbon sources by strain K1^T and *Anoxybacillus flavithermus* DSM 2641^T as assessed with API 50 CHB strips*

Carbon source	Strains	
	Strain K1^T	*A. flavithermus* DSM 2641
Glycerol	-	-
Erythritol	-	-
D-arabinose	+	+
**L-arabinose**	-	+
D-ribose	+	+
**D- xylose**	-	+
L-xylose	-	-
D-adonitol	-	-
Methyl-βD-xylopyranoside	-	-
**D-galactose**	+	-
D-glucose	+	+
**D-fructose**	+	-
**D-mannose**	-	+
**L-sorbose**	-	+
**L-rhamnose**	-	+
Dulcitol	-	-
Inositol	-	-
D-mannitol	+	+
**D-sorbitol**	-	+
Methyl-αD-mannopyranoside	-	-
Methyl-αD-glucopyranoside	-	-
N-acetylglucosamine	-	-
Amygdaline	-	-
Arbutine	-	-
Esculine	+	+
Salicin	-	-
**D-Cellobiose**	-	+
D-Maltose	+	+
D-Lactose	-	-
D-Melibiose	+	+
D-Zaharose	+	+
**D-Trehalose**	+	-
Inulin	-	-
D-Melecitose	-	-
D-Raffinose	+	+
**Amidon**	+	-
**Glycogen**	+	-
Xylitol	-	-
Gentiobiose	-	-
D-Turanose	+	+
**D-Lyxose**	+	-
D-Tagatose	+	+
D-Fucose	-	-
L-Fucose	-	-
D-Arabitol	-	-
**L-Arabitol**	-	+
**Potasium gluconate**	+	-
**2-keto-gluconate**	+	-
**5-keto-gluconate**	-	+

*Differentially utilized carbon sources are highlighted.

Table S4. Fatty acid profiles of *A. karvacharensis* K1[T] and the type strains of related *Anoxybacillus* species (values are shown as percentage of the total fatty acid content for each strain). 1, Strain K1[T]; 2, *A. flavithermus* subsp. *flavithermus* DSM 2641[T] [3]; 3, *A. flavithermus* subsp. *yunnanensis* KCTC 13759[T] [4]; 4, *A. gonensis* NCIMB 13971[T] [5]; 5, *Anoxybacillus, salavatliensis* DSM 22626[T] [6]; 6, *A. bogrovensis* DSM 17956[T] [7]; 7, *A. tengchongensis* KCTC 13721[T] [8]; 8, *Anoxybacillus eryuanensis* KCTC 13720[T] [8]; 9, *A. pushchinoensis* DSM 12423[T] [3,9]; 10, *A. kestanbolensis* NCIMB 13971[T] [10]; 11, *A. rupiensis* DSM 17127[T] [11].

Fatty acid	Strains										
	1	2	3	4	5	6	7	8	9	10	11
$C_{12:0}$	-	-	-	-	-	-	-	-	6.90	-	-
$C_{14:0}$	1.90	1.96	9.2	1.18	5.19	0.40	1.0	1.80	7.30	1.29	0.30
$C_{14:0}$ iso	0.25	-	1.3	1.25	-	0.25	4.0	0.80	-	0.88	-
$C_{14:0}$ iso 3-OH	0.31	-	-	-	-	-	-	-	-	-	-
$C_{15:0}$	-	1.18	14.0	1.12	0.42	0.40	0.30	0.30	0.90	1.11	0.30
$C_{15:0}$ iso	52.02	54.85	14.5	65.19	46.91	40.10	60.5	60.70	38.70	68.62	53.0
$C_{15:0}$ anteiso	2.18	4.02	9.5	2.64	2.81	1.80	2.80	3.50	2.0	3.56	1.60
$C_{15:0}$ iso 2-OH	-	-	-	-	-	-	-	-	0.30	-	-
$C_{16:0}$	13.15	11.13	33.4	2.38	19.41	6.30	2.40	3.90	14.50	3.47	5.40
$C_{16:0}$ iso	3.83	2.97	8.1	5.99	1.23	3.90	5.90	2.20	0.30	6.37	2.0
$C_{16:0}$ 10-methyl	-	-	-	-	-	-	-	-	0.90	-	-
$C_{16:1}$	-	-	-	-	-	-	-	-	2.60	-	-
$C_{17:0}$	0.48	-	7.1	-	-	-	2.10	2.40	0.50	-	-
$C_{17:0}$ iso	19.09	17.74	2.9	11.96	15.80	36.10	7.0	8.50	0.80	9.54	33.60
$C_{17:0}$ anteiso	4.62	6.15	-	3.29	5.33	8.98	-	-	0.10	3.69	3.90
$C_{17:0}$ anteiso A	-	-	-	0.82	-	-	-	-	-	-	-
$C_{17:1}$ $\omega 5c$	-	-	-	2.63	050	-	-	-	-	0.59	-
$C_{18:0}$	0.77	-	-	-	0.31	0.50	-	-	10.40	-	-
$C_{18:2}$	-	-	-	-	-	-	-	-	2.20	-	-
$C_{18:1}$ $\delta 9$	-	-	-	-	0.23	-	-	-	4.30	-	-
$C_{18:1}$ $\omega 9c$	1.01	-	-	-	-	-	-	-	-	-	-
$C_{18:1}$ $\delta 1$	-	-	-	-	-	-	-	-	1.0	-	-
$C_{19:0}$ iso	-	-	-	-	-	0.50	-	-	-	-	-
$C_{20:0}$	-	-	-	-	-	-	-	-	0.60	-	-

-, not detected

REFERENCES

1. **Schaeffer AB, Fulton MD**. A Simplified method of staining endospores. *Science* 1933; 77(1990):194.
2. **Meier-Kolthoff JP, Auch AF, Klenk H-P, Göker M.** Genome sequence-based species delimitation with confidence intervals and improved distance functions. *BMC Bioinformatics* 2013;14:60
3. **Pikuta E, Lysenko A, Chuvilskaya N, Mendrock U, Hippe H et al.** *Anoxybacillus pushchinensis* gen. nov., sp nov., a novel anaerobic, alkaliphilic, moderately thermophilic bacterium from manure, and description of *Anoxybacillus falvithermus* comb. nov. *Int J Syst Evol Microbiol* 2000;50:2109-2117.
4. **Dai J, Liu Y, Lei Y, Gao Y, Han F et al.** A new subspecies of *Anoxybacillus flavithermus* ssp. *yunnanensis* ssp. nov. with very high ethanol tolerance. *FEMS Microbiol Lett* 2011;320(1):72-78.
5. **Belduz AO, Dulger S, Demirbag Z**. *Anoxybacillus gonensis* sp. nov., a moderately thermophilic, xylose-utilizing, endospore-forming bacterium. *Int J Syst Evol Microbiol* 2003;53:1315-1320.
6. **Cihan AC, Ozcan B, Cokmus C.** *Anoxybacillus salavatliensis* sp. nov., an α-glucosidase producing, thermophilic bacterium isolated from Salavatli, Turkey. *JBM* 2011;51:136-146.
7. **Atanassova M, Derekova A, Mandeva R, Sjöholm C, Kambourova M**. *Anoxybacillus bogrovensis* sp. nov., a novel. thermophilic bacterium isolated from a hot spring in Dolni Bogrov, Bulgaria. *Int J Syst Evol Microbiol* 2008;58:2359-2362.
8. **Zhang CM, Huang XW, Pan WZ, Zhang J, Wei KB et al.** *Anoxybacillus tengchongensis* sp. nov. and *Anoxybacillus eryuanensis* sp. nov., facultatively

anaerobic, alkalitolerant bacteria from hot springs. *Int J Syst Evol Microbiol* 2011;61:118-122.

9. **Pikuta E, Cleland D, Tang J.** Aerobic growth of *Anoxybacillus pushchinoensis* K1^T: emended descriptions of *A. pushchinoensis* and the genus *Anoxybacillus*. *Int J Syst Evol Microbiol* 2003;53:1561-1562.

10. **Dulger S, Demirbag Z, Belduz AO**. *Anoxybacillus ayderensis* sp. nov. and *Anoxybacillus kestanbolensis* sp. nov. *Int J Syst Evol Microbiol* 2004;54:1499-1503.

11. **Derekova A, Sjoholm C, Mandeva R, Kambourova M**. *Anoxybacillus rupiensis* sp. nov., a novel thermophilic bacterium isolated from Rupi basin (Bulgaria). *Extremophiles* 2007;11(4):577-583.
